# 345. Performance Evaluation of the LifeScale AST Rapid Automated Antimicrobial Susceptibility System

**DOI:** 10.1093/ofid/ofac492.423

**Published:** 2022-12-15

**Authors:** James W Snyder, Nadia Chaudry, Wesley Hoffman

**Affiliations:** University of Louisville, Louisville, KY, Kentucky; University of Louisville, Louisville, KY, Kentucky; Jewish Hospital; UofL Healthcare, Louisville, Kentucky

## Abstract

**Background:**

Reducing the time for antimicrobial susceptibility test (AST) results directly from positive blood cultures is essential for managing patients with suspected sepsis/bacteremia caused by the most predominate Gram-negative bacteria. The aim of this study was to evaluate the LifeScale AST system using a custom LifeScale 96-well broth microdilution panel. The system employs a microfluidic sensor that detects and measures the mass of individual organisms at high throughput with MIC results available within 4-6 hours.

**Methods:**

Fresh blood cultures detected as positive by a continuous monitoring blood culture system and confirmed by Gram stain for Gram-negative rods were enrolled in the study. If a mixed Gram stain was observed, the sample was ineligible for the study. Testing was performed within 12 hours of the blood culture being flagged as positive. LifeScale performance was evaluated by comparing MICs and interpretive results to the MicroScan Walkaway 96. The following metrics were assessed: Essential Agreement (EA), Category Agreement (CA), Very Major Discrepancy (VM), Major Discrepancy (M), and Minor Discrepancy (Min).

**Results:**

A total of 65 samples were tested that met criteria for LifeScale (intended) claimed species: *E. coli* (N = 45), *Klebsiella pneumoniae* (N = 9), *Pseudomonas aeruginosa* (N = 8), *Klebsiella aerogenes* (N = 2), and one isolate each of *Acinetobacter lwoffi, Acinetobacter* species, and *Klebsiella oxytoca.* Three organisms, 2 *E. coli,* one *P. aeruginosa,* were excluded due to insufficient growth or amplitude error. The average time to results was 4.31 hours. Following resolution of discrepant AST results, there was 97.92% final agreement between LifeScale and MicroScan Walkaway 96, 36% categorical agreement, no very major discrepancies, and 0.16% major discrepancies.

**Conclusion:**

The LifeScale system provided reliable AST results for Gram-negative organisms directly from positive blood cultures with minimal hands-on time (approximately less than 8 minutes), allowing for more rapid antimicrobial management compared to standard methods. This is the first AST system that detects and measures the mass of each organism compared to traditional growth-based AST methods. Results for additional organisms will be provided once development is complete.

**Disclosures:**

**All Authors**: No reported disclosures.

